# TiO_2_ Nanocoatings with Controllable Crystal Type and Nanoscale Topography on Zirconia Implants to Accelerate Bone Formation

**DOI:** 10.1155/2022/8650659

**Published:** 2022-04-12

**Authors:** Nan Li, Zhichao Liu, Guanqi Liu, Zhi Wang, Xianwei Guo, Chuanbin Guo, Jianmin Han

**Affiliations:** ^1^Department of Dental Materials, National Engineering Research Center of Oral Biomaterials and Digital Medical Devices, NMPA Key Laboratory for Dental Materials, Peking University School and Hospital of Stomatology, Beijing 100081, China; ^2^School of Materials Science and Engineering, Beijing Institute of Technology, Beijing 100081, China; ^3^Second Clinical Division, School and Hospital of Stomatology, Peking University, Beijing 100081, China; ^4^College of Materials Sciences and Engineering, Key Laboratory of Advanced Functional Materials, Education Ministry of China, Beijing University of Technology, Beijing 100124, China; ^5^Department of Oral and Maxillofacial Surgery, Peking University School and Hospital of Stomatology, Beijing 100081, China

## Abstract

In dentistry, zirconia implants have emerged as a promising alternative for replacing missing teeth due to their superior aesthetic performance and chemical stability. To improve the osseointegration of zirconia implants, modifying their surface with hierarchical micro/nanotopography and bioactive chemical composition are two effective ways. In this work, a microscale topography was prepared on a zirconia surface using hydrofluoric acid etching, and then a 50 nm TiO_2_ nanocoating was deposited via atomic layer deposition (ALD). Subsequently, an annealing treatment was used to transform the TiO_2_ from amorphous to anatase and simultaneously generate nanoscale topography. Various investigations into the coating surface morphology, topography, wettability, and chemical composition were carried out using scanning electron microscopy, white light interferometry, contact-angle measurement, X-ray diffraction, and X-ray photoelectron spectroscopy. In addition, in vitro cytocompatibility and osteogenic potential performance of the coatings were evaluated by human bone marrow mesenchymal stem cells (hBMSCs), and in vivo osseointegration performance was assessed in a rat femoral condyle model. Moreover, the possible mechanism was also investigated. The deposition of TiO_2_ film with/without annealing treatment did not alter the microscale roughness of the zirconia surface, whereas the nanotopography changed significantly after annealing. The in vitro studies revealed that the anatase TiO_2_ coating with regular wavelike nanostructure could promote the adhesion and proliferation of osteoblasts and further improve the osteogenic potential in vitro and osseointegration in vivo. These positive effects may be caused by nanoscale topography via the canonical Wnt/*β*-catenin pathway. The results suggest that using ALD in combination with annealing treatment to fabricate a nanotopographic TiO_2_ coating is a promising way to improve the osteogenic properties of zirconia implants.

## 1. Introduction

Zirconia dental implants have been a promising candidate for replacing missing teeth in dentistry due to their outstanding aesthetic performance and inability to release metal ions, especially as compared with titanium implants [1–5]. It is generally believed that the clinical success of dental implants is directly related to their structural and functional integration with surrounding bone tissue, which is known as osseointegration [6]. Therefore, various surface modification methods, aiming to improve osseointegration of zirconia implants, have been investigated by many researchers [7]. However, since zirconia is bioinert, brittle, and relatively hard, it has proved difficult to modify the surface of zirconia implants, which has greatly limited their clinical use. Titanium implants, on the other hand, perform well in this regard and have been successfully used in clinical practice for decades. Their osseointegration ability is attributed to their surface structure and chemical composition after surface modification [8].

Various methods have been used on commercial titanium implants to modify the surface topography to obtain a hierarchical micro/nanostructure, such as sandblasting, acid etching, and anodizing [9]. Besides enlarging the area of bone-implant integration to reinforce biomechanical interlocking, previous studies have reported that a hierarchical micro/nanostructure can promote osteoblast adhesion, proliferation, and differentiation in vitro compared with a microrough surface [10–12]. Regarding the surface chemical composition, a thin oxide surface layer (∼5–10 nm) can be observed on the surface of a titanium implant, and this oxide layer is stoichiometrically similar to titanium dioxide (TiO_2_). Under physiological conditions, it is negatively charged and has hydroxyl groups attached, absorbing ions and biomolecules in vivo. At the bone-implant interface zone, it is almost in direct contact with the bone tissue, only separated by an extremely thin cell-free noncalcified tissue layer [13–15].

Existing methods of modifying the surface of commercial zirconia dental implants are the same as those used to modify titanium implants, such as sandblasting and acid etching with hydrofluoric acid [16]. While these methods work well for titanium implants, in zirconia implants, these modifications leave the implant surface bioinert and without nanoscale structure, making it inferior to the titanium implant surface. Thus, several approaches, such as sol-gel and plasma spraying, have been used to generate a TiO_2_ layer on the zirconia surface to create a surface similar to the surface of a titanium implant [17–19]. Nevertheless, the TiO_2_ coatings fabricated by these conventional surface coating methods are usually of microscale thickness and bond to zirconia surfaces physically, so it is difficult to form a continuous and uniform coating on complex microstructured zirconia surfaces, leading to a high risk of failure at the bone-implant interface. Therefore, in order to obtain a surface similar to titanium implants, it is necessary to develop a more advanced method to generate a tightly bonded TiO_2_ coating on a microstructured zirconia surface and simultaneously facilitate a hierarchical micro/nanostructure.

Atomic layer deposition (ALD) is a sequential and surface chemisorbed self-limiting thin film deposition approach that is being widely developed in semiconductor processing. It is a unique technique satisfying the need for atomic layer control and conformal deposition on structures with high aspect ratios. Hence, it seems to be a suitable approach to provide a nanoscale thickness and uniform TiO_2_ coating on the surface of zirconia implants [20]. Furthermore, the crystal type and nanoscale topography of the deposited TiO_2_ nanocoating can be easily controlled with annealing treatment. TiO_2_ has three main crystal types: anatase, rutile, and brookite. Previous studies have reported that anatase TiO_2_ is most suitable for bone growth since the anatase surface has a larger amount of hydroxyl groups, which may induce a conformational change of adsorbed protein to increase the exposure of cell-binding sites [21–23]. Moreover, in line with this finding, anatase is the main crystal type found on the surfaces of anodized titanium implants [24, 25].

In the present study, a nanoscale thickness TiO_2_ coating was successfully prepared on a hydrofluoric acid etched zirconia surface using ALD. Anatase coatings with different nanoscale topographies were obtained by applying different annealing processes. The osteogenic potential of each coating was assessed via in vitro and in vivo experiments. Moreover, the possible mechanism was also investigated. To the best of our knowledge, using ALD to assemble TiO_2_ nanocoatings with controllable crystal type and nanoscale topography on microrough zirconia surfaces has never been explored before.

## 2. Materials and Methods

### 2.1. Material Preparation and TiO_2_ Coating Fabrication

First, 8 mol% Ceria- and 2 mol% Yttria-doped zirconia powder were isostatically pressed under 167 MPa and then sintered at 1450°C for 2 h in the air to final shape. In this work, zirconia samples were used in the form of disks in vitro (*φ*15 × 1.5 mm^2^) and cylindrical implants in vivo (*φ*2 × 5 mm^2^). Based on a preliminary experiment, all zirconia specimens were etched by 32% hydrofluoric acid at 80°C for 30 min before ALD to obtain the ideal surface morphology.

The resulting etched samples were given a TiO_2_ coating with a thickness of 50 nm by using an atomic layer deposition reactor (ALD, Ensure Nanotech, Labnano 9100). Titanium tetrakis (dimethylamide) (TDMAT) was used as the precursor, H_2_O (pure water) was used as the oxidant, and N2 (purity = 99.9999%) served as the carrier and purging gas under the growth temperature of 120°C. In addition, (100) p-type silicon (Si) wafers were added as assistive substrates for ellipsometry and X-ray diffraction (XRD, D8 Advance, Bruker Nano GmbH Berlin, Germany). Furthermore, the resulting samples were subjected to different annealing processes in the air using a furnace (HT-2/M/ZIRKON-120, MIHM-VOGT GMBH & CO. KG).

We carried out a preliminary experiment to determine the suitable annealing condition. One each for Si wafer and zirconia sample was used for each combination of different temperatures (300°C, 400°C, 600°C, and 800°C) and times (5 min and 1 h). The crystal type of the coatings was detected by X-ray diffraction (XRD, D8 Advance, Bruker Nano GmbH Berlin, Germany) on the Si wafers. The bone-bonding ability was evaluated by examining the apatite forming ability of the zirconia samples in a simulated body fluid (SBF, Leagene) for 7 days at 37°C [26]. According to the results, two representative annealed samples were selected as experimental groups in this study: ALD-TiO_2_-A1 was annealed at 800°C for 5 min, and ALD-TiO_2_-A2 was annealed at 300°C for 1 h. The hydrofluoric acid etched samples, denoted as HF, and the as-deposited TiO_2_ samples, denoted as ALD-TiO_2_, were used as controls.

### 2.2. Surface Characterization

Scanning electron microscopy (SEM, SU8220/Regulus8230, Hitachi, Japan) was used to detect the surface morphology of the samples. Cross-sectional mapping and line scanning were performed with an energy-dispersive X-ray spectrometer (EDS, Bruker Nano GmbH Berlin, Germany) and a Bruker FlatQuad X-ray spectrometer to detect element composition and distribution. All samples were coated with carbon (C) before observation.

Surface roughness was detected by white light interferometry (WLI, ZYGO, USA) under a 50x magnifying glass. Wettability was calculated from the contact angle of 2 *μ*L deionized water on the surface of the samples measured by using a video contact-angle measurement instrument.

The crystallinity degree of the TiO_2_ films was detected by XRD. Scanning was operated with 2*θ* in the range of 20°–55° and using CuK*α* radiation as the source at a rate of 2 /min and with 1° glancing angle. Phase analysis was performed using the standard JCPDS database.

X-ray photoelectron spectroscopy (XPS, PHI QUANTERA-II SXM, ULVAC-PHI, inc. Japan) was used to characterize the surface elemental compositions and chemical states. XPS spectra were recorded using Al K*α* radiation (1486.6 eV) as the source. A survey spectrum and high-resolution spectra for Ti2p signals were obtained for each sample. The binding energy of the C1s signal was used for spectral charge correction, and fitting analysis was performed using MultiPak Software (Gaussian multipeak fitting).

### 2.3. In Vitro Cytocompatibility Evaluation

#### 2.3.1. Cell Culture

Human bone marrow mesenchymal stem cells (hBMSCs; ATCC, USA) were cultured in alpha minimum essential medium (*α*-MEM; Hyclone, USA) containing 10% fetal bovine serum (FBS, Gibco, Australia) and 1% penicillin/streptomycin (Gibco) at a humidified atmosphere of 5% CO_2_ under 37°C. Fourth- to sixth-generation cells were seeded on the samples with a density of 3 × 104 cells/well in a 24-well tissue culture plate (Corning).

For osteogenic induction, the medium was replaced with osteogenic inducing medium (normal growth medium supplemented with 10 mM *β*-glycerophosphate (Sigma), 50 *μ*g/mL ascorbic acid (Sigma), and 10 nM dexamethasone (Sigma)) after 3 d of incubation. The medium was changed every 2 d during the culture period.

#### 2.3.2. Cell Morphology and Cytoskeleton

After 1 and 3 d of incubation, cells on each sample were fixed with 2.5% glutaraldehyde (v/v) for 30 min and dehydrated in gradient ethanol of 50%, 75%, 90%, and 99% for 15 min each. Then, the samples were dried, sprayed with Pt, and observed by SEM (Phenom ProX, Thermo Fisher Scientific).

After 1 and 3 d of incubation, cells on each sample were fixed with 4% paraformaldehyde (v/v) for 30 min, permeabilized with 0.2% Triton X-100 (v/v, Amresco, USA) for 5 min, stained with rhodamine phalloidin (Cytoskeleton Inc., USA) for 30 min and DAPI-Fluoromount-G (Southern Biotech Co., USA) for 5 min, and then observed by laser scanning confocal microscope (LSM 710, Zeiss Co., Germany).

#### 2.3.3. Cell Proliferation Evaluation

The cell proliferation on different samples was determined using cell counting kit-8 (CCK-8, Donjindo) after 1, 3, and 5 d of incubation. At each time point, the medium was refreshed with a serum-free medium containing 10% CCK-8 reagent and cultured for 1 h in a humidified atmosphere of 5% CO_2_ at 37°C. Then, 100 *μ*L supernatant of each well was transferred into a 96-well plate and measured by a microplate reader (SoftMax Pro, Molecular Devices) at a wavelength of 450 nm.

### 2.4. In Vitro Osteogenic Potential Assessment and Possible Mechanism Investigation

#### 2.4.1. Alkaline Phosphate Activity Quantification and Staining

The alkaline phosphatase (ALP) activity was determined to estimate early osteogenic differentiation of hBMSCs on different samples using an ALP activity kit (Nanjing Jiancheng Biotechnology, China). After 3 and 7 d of incubation, cells on each sample were collected using 1% Triton X-100 (v/v) and then centrifuged at 12,000 rpm, 4°C for 30 min. Next, 30 *μ*L supernatant of each well was mixed with 100 *μ*L ALP activity kit working solution, transferred into a 96-well plate, and measured by a microplate reader at a wavelength of 520 nm. The total protein concentration was also determined by a bicinchoninic acid (BCA) protein assay kit (Thermo Fisher Scientific) to enable standardization. Finally, the ALP activity value was standardized and expressed as the total protein content (U/g protein).

For ALP staining, after 7 d of incubation, cells on each sample were fixed by 99% ice ethanol for 30 min, and then, a BCIP/NBT ALP color development kit (Beyotime Biotechnology, China) was used to stain for 30 min according to the instruction manual. The samples were finally scanned by a scanner (HP Scanjet, G4050, USA).

#### 2.4.2. Alizarin Red S Staining and Quantification

Alizarin Red S staining was used to estimate the mineralized nodule formation after 21 d of incubation. The cells on each sample were fixed by 4% paraformaldehyde for 20 min, and 1% Alizarin Red S solution (pH = 4.2, Solarbio, China) was added to each well for 20 min. The excess solution was then thoroughly removed with deionized water, and the deposited calcium was recorded. For further quantification, 10% hexadecylpyridinium chloride (w/v, Sigma) was added to dissolve the red matrix sediment completely, and 100 *μ*L solution per well was transferred into a 96-well plate and measured by a microplate reader at a wavelength of 550 nm.

### 2.5. Quantitative Polymerase Chain Reaction (qPCR) Analysis

After 7 d of incubation, the total RNA was extracted using a TRIzol reagent (Invitrogen, Thermo Fisher Scientific) and reversely transcribed into cDNA using a PrimeScriptTM RT Master Mix (Takara, Biotechnology, Shiga, Japan) according to the instruction manual. Then, the 2-fold diluted cDNA was used as the template in the 20-*μ*L qPCR reactions on an ABI Q3 instrument (Applied Biosystems, USA) with SYBR Green Master (Roche). Finally, *β*-actin was used as the housekeeping gene for normalization, and gene expression was quantitatively determined by cycle threshold (Ct) values according to the ^△△^Ct method. The primer pairs used in this study are given in Table 1.

#### 2.5.1. Immunofluorescence Staining

After 7 d of incubation, the cells on different samples were fixed by 4% paraformaldehyde for 15 min, washed three times with PBS, permeabilized with 0.25% Triton-100 (v/v) for 10 min, and blocked with 0.8% bovine serum albumin (BSA, w/v, Amresco, USA). Then, the samples were incubated with primary antibodies anti-osteopontin and anti-osteocalcin (1 : 100, in 0.8% BSA, Abcam, UK) overnight at 4°C and washed with PBS for 5 min three times. Next, the samples were incubated with fluorescein-conjugated secondary antibody (1 : 100, in PBS) for 1 h away from light. Finally, DAPI was used to stain cell nuclei for 5 min, and the samples were observed by CLSM.

#### 2.5.2. ELISA

After 7 d of incubation, the cells on samples were collected to extract the total protein. The protein concentration was measured and standardized using a BCA protein assay kit. The *β*-catenin protein was inspected using a *β*-catenin ELISA kit (MEIMIAN, Jiangsu, China) according to the instruction manual. The absorbance was measured by a microplate reader at a wavelength of 450 nm.

### 2.6. In Vivo Osseointegration Study

#### 2.6.1. Surgical Implantation

Forty Sprague-Dawley (SD) rats weighing 200–220 g were used for in vivo experiments. The study was approved by the Ethics Committee of the Peking University Health Science Center, Beijing, China (Approval No.: LA2020514). Eighty zirconia cylindrical implants (*φ*2 × 5 mm^2^) were randomly assigned to four groups corresponding to HF, ALD-TiO_2_, ALD-TiO_2_-A1, and ALD-TiO_2_-A2 for in vivo study.

General anesthesia was performed using 1% pentobarbital sodium (w/v, 50 mg/kg), and the implant site was prepared ∼5 mm deep at femoral condyles using a slow-speed drill with a 2 mm round burr under continuous irrigation with 0.9% sterile saline (w/v). The cylindrical implant was inserted into the hole until the end of the implant aligned with the femoral condyle surface. After 4 and 12 weeks of healing, the bilateral femora containing cylindrical implants were harvested, with forty samples per healing period. Five samples of each group per healing period were subjected to a biomechanical (push-in) test, and the other five were used for histological analysis.

#### 2.6.2. Sequential Fluorescent Labeling

Polychrome sequential fluorescent labeling was performed to assess the bone formation and mineralization process [27]. For 4-week experimental groups, at 1 and 3 weeks after implantation, two different fluorochromes were injected intraperitoneally into the rats at a sequence of 30 mg/kg Alizarin Red S (Sigma) and 20 mg/kg calcein (Sigma). For 12-week groups, at 4, 8, and 11 weeks after implantation, three different fluorochromes were injected intraperitoneally into the rats at a sequence of 30 mg/kg Alizarin Red S (Sigma), 20 mg/kg calcein (Sigma), and 25 mg/kg tetracycline hydrochloride (Sigma).

#### 2.6.3. Push-In Test

After 4 weeks and 12 weeks of healing, femurs containing specimens were harvested and immersed in PBS with both top and bottom surfaces revealed. The implants were then loaded axially in a universal mechanical testing machine (Instron 5969, Norwood, MA, USA) using a 500 N load cell and a 1.5 mm diameter stainless-steel pushing rod with a constant speed of 0.5 mm/min. The push-in value was determined as the breaking-point load, which refers to the maximum load before a rapid decrease in the load-displacement curve.

#### 2.6.4. Histological Analysis

Five samples of each group per healing period were fixed in 10% buffered formalin for 1 week, dehydrated by ascending concentrations of ethanol, and finally embedded in polymethylmethacrylate (PMMA).

The samples were cut into longitudinal sections with a thickness of 200 *μ*m using the EXAKT system (310 CP, EXAKT). Then, the slices were ground to a thickness of about 100 *μ*m to observe polychrome fluorescent labeling by CLSM. The excitation/emission wavelengths used to visualize the chelating fluorochromes were 543/600–640 nm (Alizarin Red S, red), 488/500–550 nm (calcein, green), and 405/560–590 nm (tetracycline hydrochloride, yellow) to assess the time course of bone formation.

After fluorescence observation, the slices were further ground and polished to a final thickness of about 50 *μ*m and stained with Stevenel's blue/van Gieson's picrofuchsin to stain the calcified bone red and the soft tissue blue. The images were acquired by Multiple Image Alignment (MIA). The BIOQUANT OSTEO 2019 system (BIOQUANT Image Analysis Corporation, Nashville, TN) was used to measure the bone-to-implant contact (BIC) value and the bone volume/total volume (BV/TV) ratio.

### 2.7. Statistical Analysis

The data were expressed as mean ± standard deviation (SD). All the in vitro studies involved three independent experiments, and each data point represented three replicate measurements. GraphPad Prism 9.0 (GraphPad Software, Inc., USA) was used to analyze quantitative data using one-way analysis of variance (ANOVA) with Tukey-Kramer multiple comparison posttest. *p* < 0.05 was considered to be statistically significant.

## 3. Results and Discussion

### 3.1. Surface Characterization

**Figure 1:**
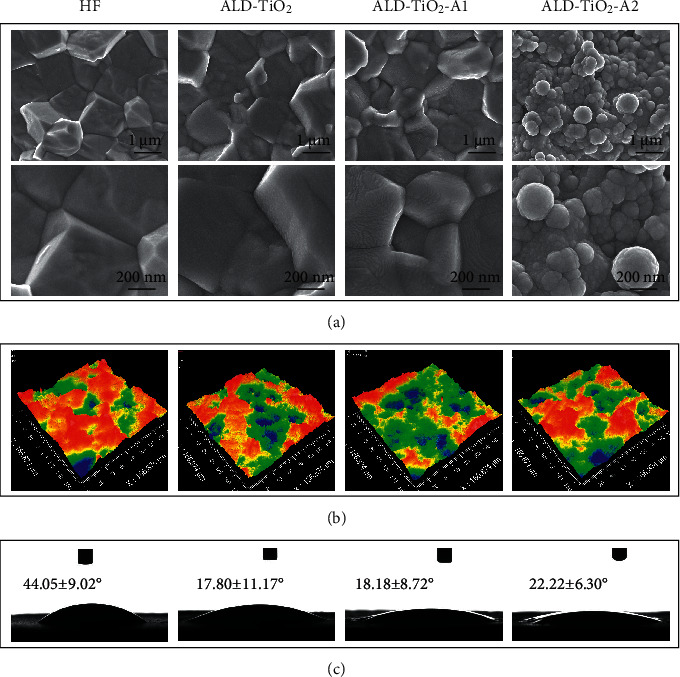
(a) Surface morphology assessed by scanning electron microscopy (SEM) at different magnifications and (b) white light interferometry (WLI). (c) Water contact angle measurements on different samples.

Figure 1(a) shows the SEM images of the sample surfaces at different magnifications. The TiO_2_ coating closely and uniformly adheres to the surface of zirconia. The general morphology has no significant differences before and after ALD treatment. A regular wavelike nanopattern and nanoscale sphere-like structures can be seen on the ALD-TiO_2_-A1 and ALD-TiO_2_-A2, which were treated with different annealing processes.

The WLI data (Figure 1(b)) show that the roughness (Sa) (Table 2) of the four groups is around 1.3 *μ*m, and there is no significant difference among them. This roughness can be considered as a moderate roughness that generates the best osseointegration capacity and provides mechanical interlocking shortly after implantation [10, 28]. In addition, the 3D roughness parameters Sq, which represents the standard deviation of height values, and Sz, which represents the average difference between the highest peaks and lowest valleys, were determined to gain more detailed surface topography information [29]. The four groups have no statistically significant differences in these two parameters. These results indicate the success of hydrofluoric acid etching, and the findings are in line with previous studies [30].

Figure 1(c) shows that the surface wettability significantly improved after TiO_2_ coating, whereas there was no significant difference among ALD-TiO_2_, ALD-TiO_2_-A1, and ALD-TiO_2_-A2.

**Figure 2:**
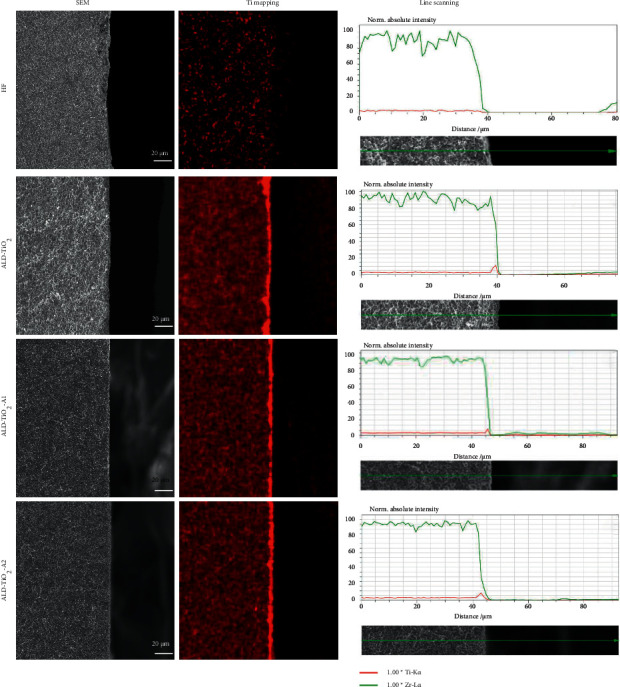
Cross-sectional mapping results of different samples.

The cross-sectional mapping and line scan EDS of Ti depicted in Figure 2 clearly show that the TiO_2_ coatings with/without annealing all have a thin and uniform distribution on the zirconia surface.

**Figure 3:**
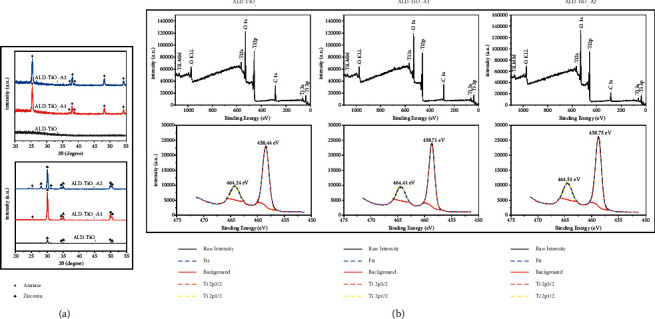
(a) XRD diffraction patterns of TiO_2_ coatings deposited on Si wafers (top) and zirconia disks (bottom). (b) Full-scan survey spectra and high-resolution XPS spectra of different coatings.

The XRD spectra of ALD-TiO_2_, ALD-TiO_2_-A1, and ALD-TiO_2_-A2 on the zirconia disc and Si wafer are consistent (Figure 3(a)). The as-deposited TiO_2_ coating is in an amorphous form, whereas the two annealing groups, ALD-TiO_2_-A1 and ALD-TiO_2_-A2, are both in the anatase phase and have similar crystallinity of 40% (quantitated by XRD results on Si wafers).

Figure 3(b) shows the XPS results. Ti, O, and C are detected on the surface. The high-resolution region spectrum of Ti 2p shows double peaks, and the difference value is between 5.7 eV and 5.8 eV, which can be designated as the Ti^4+^ oxidation state according to the XPS handbook, suggesting the existence of TiO_2_ [31]. The binding energies of Ti 2P3/2 were 458.71 eV for ALD-TiO_2_-A1, 458.75 eV for ALD-TiO_2_-A2, and 458.44 eV for ALD-TiO_2_. The results indicated that the annealing treatment increased the binding energy of the Ti^4+^ oxidation state.

Taken together, we have successfully fabricated the microstructured zirconia surface using hydrofluoric acid, and a densely and uniformly TiO_2_ nanocoating was also successfully deposited on the zirconia surface by ALD. The subsequent annealing procedure changed the coating's crystal type and nanoscale topography. The present study successfully prepared the HF control group, the ALD-TiO_2_ group with amorphous TiO_2_ coating, the ALD-TiO_2_-A1 group with regular wavelike nanopattern, and the ALD-TiO_2_-A2 group with nanoscale sphere-like structures. In addition, since the two anatase TiO_2_ coatings have the same chemical composition and crystallinity, the nanoscale structure may play a predominant role in determining specific cellular responses.

### 3.2. In Vitro Cytocompatibility

Initial cell adhesion is the first step of cell–material interactions, which is usually responsible for cell function and ultimate tissue integration, while cell proliferation is closely related to the amount of new bone formation [32–34]. Therefore, the cell adhesion and proliferation of hBMSCs are vital factors that contribute to the bone formation around the implants.

**Figure 4:**
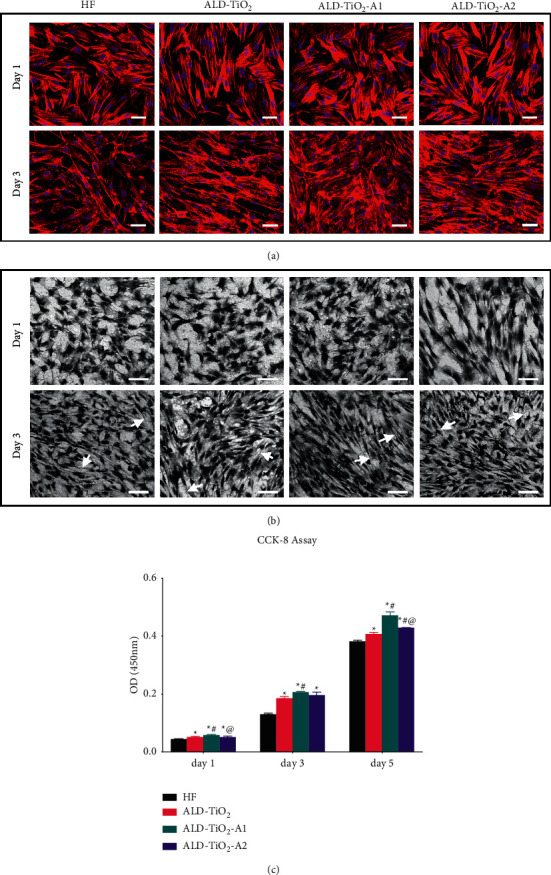
Morphology of hBMSCs after 1 and 3 days of incubation on different samples investigated by (a) CLSM and (b) SEM. The arrows indicated the adhered filopodia. (c) Proliferation of hBMSCs on different samples after 1, 3, and 5 days. Bar = 100 *μ*m. Data were shown as mean ± SD. *p* > 0.05 ^*∗*^*p* < 0.05 compared to the HF; ^#^*p* < 0.05 compared to the ALD-TiO_2_; ^@^*p* < 0.05 compared to the ALD-TiO_2_-A1.

For cell adhesion, Figure 4(a) shows the cytoskeletons (F-actin) of hBMSCs grown on the sample on day 1 and day 3. On day 1, hBMSCs exhibited immature F-actin on all samples. After culturing for 3 d, the hBMSCs grown on all samples proliferated to cover the surface completely. Compared with HF, hBMSCs on the surfaces of coated groups showed more F-actin filament aggregation sites, and those on the ALD-TiO2-A1 surface exhibited more evident cell–cell contacts.

In addition, the morphologies of hBMSCs on all samples after 1 and 3 d were further investigated by SEM (Figure 4(b)). After culturing for 1 d, hBMSCs grown on all samples demonstrated a polygonal shape, and those on the surface of ALD-TiO_2_-A2 exhibited more extended lamellipodia. After 3 d of culture, cells aggregated and occupied almost the entire surface. Additionally, hBMSCs grown on the ALD-TiO_2_-A1 surface exhibited more elongated filopodia compared with HF, ALD-TiO_2_, and ALD-TiO_2_-A2.

The CCK-8 assay was performed after 1, 3, and 5 d to investigate the cell proliferation on the surface of different samples (Figure 4(c)). The increased OD value indicated that hBMSCs on all surfaces were proliferating over time. Compared with HF, significantly increased cell proliferation was observed on the surfaces of coated groups at each time point, especially on ALD-TiO_2_-A1.

Combining the results of CCK-8 with SEM and CLSM images, we find that the surfaces of coated groups have better cytocompatibility than HF, which may be due to promoted surface wettability. In addition, in the three coated groups, hBMSCs grown on the surface of ALD-TiO_2_-A1 display better overall results, suggesting that the regular wavelike nanopatterned topography may provide a more favorable cell environment for cell adhesion and proliferation.

### 3.3. In Vitro Osteogenic Potential

In order to enhance osseointegration, the surface of the zirconia implant should have effective osteogenic activity. The ALP activity, calcium deposition amount, protein expression, and mRNA expression of osteogenic-related markers were detected to measure the osteogenic capability of hBMSCs on different samples.

**Figure 5:**
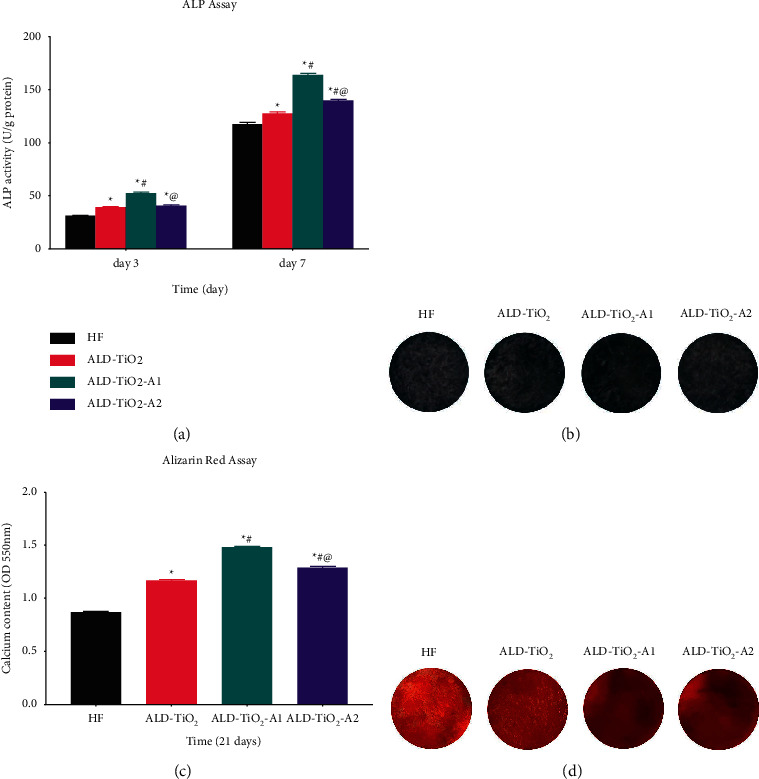
(a) Quantitative determination of ALP activity on day 3 and day 7 and (b) representative ALP staining on day 7. (c) Quantitative determination of calcium deposition for 21 d and (d) representative Alizarin Red S staining. Data were shown as mean ± SD.  ^*∗*^*p* < 0.05 compared to the HF; ^#^*p* < 0.05 compared to the ALD-TiO_2_; ^@^*p* < 0.05 compared to the ALD-TiO_2_-A1.

As an important early osteogenic marker [35], ALP activity was measured on days 3 and 7 of osteogenic induction. As shown in Figure 5(a), ALP activity increased with the culture time. At each time point, ALD-TiO_2_-A1 exhibited the highest ALP activity, and HF showed the lowest ALP activity. There were significant differences between the groups, except ALD-TiO_2_ and ALD-TiO_2_-A2 on day 3. The ALP staining on day 7 also displayed a similar result (Figure 5(b)). These results indicate that TiO_2_ nanocoatings have a positive effect on osteogenic differentiation.

The amount of calcium deposition is the result of osteogenic differentiation in the later stage [36]. After 21 d of osteogenic induction, Alizarin Red S staining and quantification were performed to determine the matrix mineralization of hBMSCs. As shown in Figure 5(d), Alizarin Red S staining on the surfaces of anatase TiO_2_ coated groups exhibited broader and denser than that on HF and ALD-TiO_2_ surfaces. However, the differences between the staining results of ALD-TiO_2_-A1 and ALD-TiO_2_-A2 were challenging to see with naked eyes. The quantitative results (Figure 5(c)) showed that the amounts of calcium deposition on the surfaces of the coated groups were significantly higher than that on the HF surface. Remarkably, the calcium deposition on ALD-TiO_2_-A1 was the largest, consistent with the ALP quantification and staining results.

**Figure 6:**
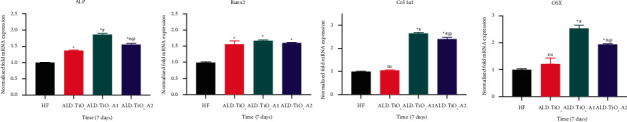
The mRNA expression level of ALP, Runx2, Col 1*α*1, and OSX detected by qPCR after 7 d. Data were shown as mean ± SD.  ^*∗*^*p* < 0.05 compared to the HF; ^#^*p* < 0.05 compared to the ALD-TiO^2^; ^@^*p* < 0.05 compared to the ALD-TiO_2_-A1; ns, no significant statistic difference (*p* > 0.05).

To further investigate the influences of different sample surfaces on the osteogenic differentiation of hBMSCs at the molecular level, the mRNA expressions of osteogenic-related genes, including ALP, Runt-related transcription factor 2 (Runx2), Collagen Type I Alpha 1 (Col 1*α*1), and Osterix (OSX), were quantified using qPCR after 7 d of osteogenic induction. The results are displayed in Figure 6.

ALP is an early marker of osteogenic differentiation. Runx2 is considered to be an osteoblast-specific transcription factor that is necessary for inducing osteoblast differentiation. When increased in mesenchymal cells, it can trigger the osteogenic gene expression program and activate the expression of downstream osteogenic genes, such as ALP, Col 1*α*1, OCN, and OPN [37]. Col 1*α*1 is a marker related to the deposition of extracellular matrix [38]. OSX is a zinc finger-containing transcription factor essential for the maturation of osteoblasts [39]. In this study, hBMSCs cultured on the surfaces of the three coated groups induced significantly higher expressions of ALP and Runx2 than those on HF, suggesting that the TiO_2_ coatings can significantly promote osteoblast differentiation. The expression levels of Col 1*α*1 and OSX were statistically higher on the anatase TiO_2_ coated surfaces than on HF and ALD-TiO_2_. Among the three coated groups, the expression levels of ALP, Col 1*α*1, and OSX displayed upregulation on the anatase TiO_2_ coated surfaces when compared to the amorphous TiO_2_ coated surface, and their expression levels were higher on ALD-TiO_2_-A1. However, the expression level of Runx2 exhibited no statistical difference among the three coated groups. The result might be related to the selected time point, as Runx2 is an early regulator of osteogenic differentiation whose expression shows a trend of increasing and then decreasing with the maturation of osteoblasts [40–42].

**Figure 7:**
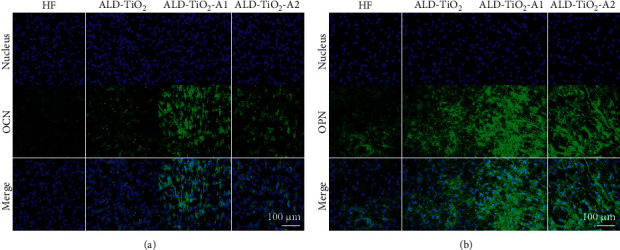
Protein expression level of OCN (a) and OPN (b) detected by immunofluorescence after 7 d.

Immunofluorescence staining further verified the effects of surface topography and chemical composition on the osteogenic differentiation of hBMSCs at the protein level. Osteocalcin (OCN), the most abundant noncollagenous protein in the mineralized bone matrix, is involved in the mineralization process, and its release is related to the initiation of mineralization [43]. Meanwhile, osteopontin (OPN) is also an essential component of the mineralized extracellular matrix of bone and teeth [44]. The immunofluorescence staining results of OCN and OPN are shown in Figure 7. After 7 d of osteogenic induction, hBMSCs on all the surfaces exhibited recognizable OCN-positive and OPN-positive staining, and stronger fluorescent intensity was seen on coated groups' groups, especially on ALD-TiO_2_-A1.

Summarizing the results above, we believe that TiO_2_ nanocoatings have a positive effect on cell adhesion, proliferation, and osteogenic differentiation, and anatase TiO_2_ nanocoatings perform even better. Moreover, between the two anatase TiO_2_ nanocoatings, ALD-TiO_2_-A1, which has a regular wavelike nanostructure, exhibits outstanding results compared with ALD-TiO_2_-A2, indicating the superiority of its nanoscale topography.

### 3.4. In Vivo Osseointegration

To evaluate the effects of different sample surfaces on osseointegration in vivo, a push-in test was performed to measure the bone-implant bond strength, while histological analysis was performed to characterize the bone regeneration on the bone-implant interface.

**Figure 8:**
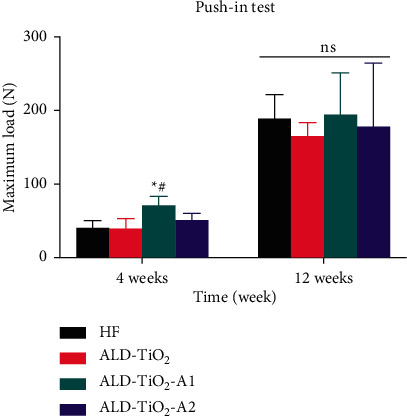
Push-in strength of the implants at 4 and 12 weeks after implantation. Data were shown as mean ± SD.  ^*∗*^*p* < 0.05 compared to the HF; ^#^*p* < 0.05 compared to the ALD-TiO_2_; ^@^*p* < 0.05 compared to the ALD-TiO_2_-A1; ns, no significant statistic difference (*p* > 0.05).

As shown in Figure 8, the push-in strength of all samples increased continuously during the observation period. ALD-TiO_2_-A1 had a statistically higher push-in strength than HF and ALD-TiO_2_ at 4 weeks after implantation, whereas there was no significant difference among all the groups at 12 weeks, indicating that the anatase TiO_2_ nanocoating with regular wavelike nanostructure can induce stronger biomechanical bonding between the implant and bone in the early stage.

**Figure 9:**
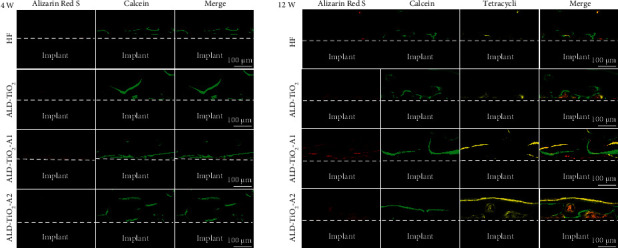
Polychrome sequential fluorescent labels at 4 weeks and 12 weeks after implantation in rat femoral condyle models: 4 weeks (a) Alizarin Red S (red, 1 week) and calcein (green, 3 weeks); 12 weeks (b) Alizarin Red S (red, 4 weeks), calcein (green, 8 weeks), and tetracycline hydrochloride (yellow, 11 weeks).

The process of new bone formation and mineralization around the implants was observed by different types of fluorochrome labeling at specific time intervals of 1, 3, 4, 8, and 11 weeks. As shown in Figure 9, in the 4-week experimental groups, Alizarin Red S red was almost undetectable at 1 week, which only exhibited a slight red fluorescence area adjacent to the implants of ALD-TiO_2_-A1. At 3 weeks, calcein green was found deposited onto a broader area extending along the ALD-TiO_2_-A2 surface. In the 12-week experimental groups, Alizarin Red S red was seen to be deposited onto a border area close to the ALD-TiO_2_-A2 surface at 4 weeks. Similar trends for calcein green and tetracycline yellow were observed at 8 weeks and 11 weeks, respectively. The results are consistent with those of the 4-week healing period. The calcein green and tetracycline yellow fluorescence were found to spread throughout the entire region from the implant surface toward the external area, and ALD-TiO_2_-A1 exhibited the most intense and widely distributed fluorescent lines. Moreover, the gaps between different fluorescent lines are more distinctive on the ALD-TiO_2_-A1 surface, indicating a faster bone formation rate compared to the other groups.

**Figure 10:**
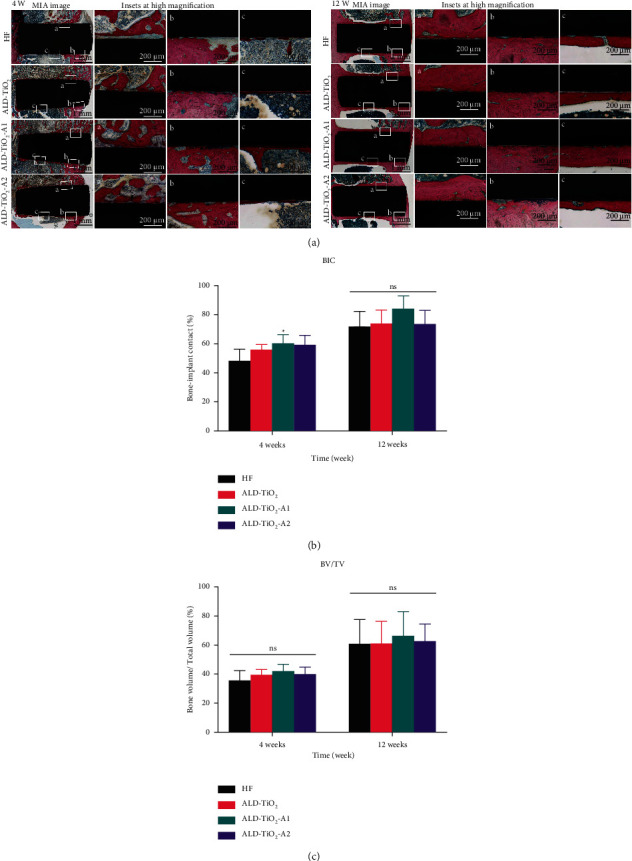
(a) Stevenel's blue/van Gieson's picrofuchsin staining images at 4 weeks (left) and 12 weeks (right) after implantation. (b) The BIC values and (c) the BV/TV ratios of HF, ALD-TiO_2_, ALD-TiO_2_-A1, and ALD-TiO_2_-A2 at 4 weeks and 12 weeks after implantation. Data were shown as mean ± SD.  ^*∗*^*p* < 0.05 compared to the HF; ^#^*p* < 0.05 compared to the ALD-TiO2; ^@^*p* < 0.05 compared to the ALD-TiO_2_-A1; ns, no significant statistic difference (*p* > 0.05).

In order to evaluate the bone response to different sample surfaces in the early and late healing stage, hard tissue sections stained with Stevenel's blue/van Gieson's picrofuchsin were performed on the bone-implant interface for histological analysis (Figure 10(a)). After 4 weeks of healing, the newly formed bone bonded tightly and directly to the surface of ALD-TiO_2_, ALD-TiO_2_-A1, and ALD-TiO_2_-A2, whereas the HF implant was mostly surrounded by a layer of fibrous tissue (blue), separating the bone tissues from the surface. After 12 weeks, large amounts of newly formed and continuous mineralized bone were observed in the cortico-cancellous bone around all the implants.

Figure 10(b) shows the BIC values. For the 4-week healing period, the BIC value of ALD-TiO_2_-A1 was 60.20 ± 6.06%, significantly higher than HF (48.40 ± 8.081%, *p* < 0.05), but not significantly different compared with ALD-TiO_2_ (56.20 ± 3.27%) and ALD-TiO_2_-A2 (59.33 ± 6.35%). Moreover, the BIC values increased at 12 weeks, but there was no statistically significant difference among all the groups (HF: 72.00 ± 10.27%; ALD-TiO_2_: 74.00 ± 9.22%; ALD-TiO_2_-A1: 84.20 ± 8.67%; ALD-TiO_2_-A2: 72.40 ± 7.76%).

Figure 10(c) shows the BV/TV values. ALD-TiO_2_-A1 exhibited the highest mean value, followed by ALD-TiO_2_-A2, ALD-TiO_2_, and HF. However, there was no significant difference among all the groups for the 4- and 12-week healing periods.

Taking the upper results together, we find that ALD-TiO2-A1 exhibited stronger bone-implant bond strength and higher bone regeneration capacity than the other three groups (especially the HF control) at 4 weeks after implantation. When it comes to 12 weeks, the differences among the four groups were not statistically significant. These results suggest that the anatase TiO_2_ coating with a regular wavelike nanostructure can facilitate the early osseointegration of zirconia implants, which is beneficial to shorten the implantation period and has clinical significance [45].

In general, the results of in vivo studies are congruent with the above in vitro findings, indicating that the ALD-TiO_2_-A1 surface induces more new bone formation and enhances bone-implant integration, especially in the early healing stage.

### 3.5. Activation of the Canonical Wnt/*β*-Catenin Pathway

Summarizing the above results, the anatase and nanoscale structured TiO_2_ coating surfaces can improve the osteogenic potential in vitro and in vivo. The ALD-TiO_2_-A1 surface, which has a regular wavelike nanopattern, can improve the osteogenic potential more than the ALD-TiO_2_-A2 surface, which has a nanoscale sphere-like structure. Since the two surfaces share the same chemical composition and crystallinity, we speculate that the difference in their performance is caused by the difference in their nanoscale topography.

**Figure 11:**
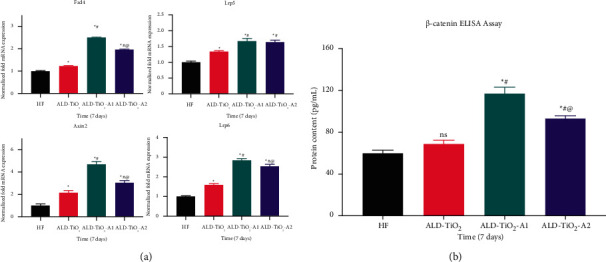
(a) The mRNA expression level of Fzd4, Axin2, Lrp5, and Lrp6 detected by qPCR after 7 days. (b) The *β*-catenin protein expression detected by ELISA after 7 days. Data were shown as mean ± SD,  ^*∗*^*p* < 0.05 compared to the HF; ^#^*p* < 0.05 compared to the ALD-TiO2; ^@^*p* < 0.05 compared to the ALD-TiO_2_-A1; ns, no significant statistic difference (*p* > 0.05).

To explore the possible mechanism, mRNA expression of Wnt/*β*-catenin signal pathway-related genes Frizzled 4 (Fzd4), Axis inhibition protein 2 (Axin2), low-density lipoprotein receptor-related protein 5 (Lrp5), and low-density lipoprotein receptor-related protein 6 (Lrp6) were quantified using qPCR after 7 d of osteogenic induction. As shown in Figure 11(a), the transcription levels of these four genes were significantly upregulated on ALD-TiO_2_-A1 surfaces compared with HF and ALD-TiO_2_. In comparison to ALD-TiO_2_-A2, the expressions of Fzd4, Axin2, and Lrp6 mRNAs were higher on ALD-TiO_2_-A1. The result is consistent with the previous finding of higher Lrp6 expression in MG63 cultured on micropitted/nanotubular surface topographies [46]. Fzd4 has been reported as one of the most intensely modulated genes by nanotopography [47]. Moreover, we also determined the protein expression of *β*-catenin, which is known as the key molecular node of the Wnt/*β*-catenin signaling pathway [48]. As expected, hBMSCs cultured on ALD-TiO2-A1 surfaces revealed significantly higher levels of *β*-catenin than other groups (Figure 11(b)).

**Figure 12:**
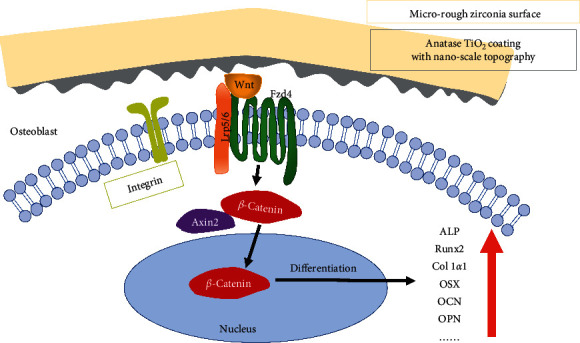
orking model of the mechanism of nanoscale topography mediates osteoblast differentiation via the canonical Wnt/*β*-catenin pathway.

The canonical Wnt/*β*-catenin signaling pathway is considered to play an essential role in osteoblast differentiation, maturation, and bone mass generation [49]. Moreover, previous studies have shown that it participates in the influence of nanoscale topography on osteoblast differentiation [46, 47, 50]. Here, our findings suggest that the nanoscale topography of the ALD-TiO_2_-A1 surface appears to play an important role in promoting osteogenic differentiation of hBMSCs via the canonical Wnt/*β*-catenin pathway. We propose a working model based on the analysis above, as shown in Figure 12.

The process of bone regeneration includes cell adhesion, spread, proliferation, differentiation, and extracellular matrix mineralization, which is regulated by the surface topography and chemical composition of the material [51]. Accordingly, the clinical success of titanium implants benefits from their hierarchical micro/nanosurface structure and the thin oxide surface layer. The current commercial zirconia implants are most treated by sandblasting and etching, generating microrough surfaces without nanoscale structure. The overall survival and success rates of zirconia implants are low, indicating that the effect of the current surface treatment method is not ideal [28]. Hence, in this study, we prepared a coating on the zirconia implant with the same chemical composition and structure as the titanium implant. The purpose of the coating was to combine the aesthetic advantage of zirconia and the superior osseointegration of titanium implants.

The TiO_2_ nanocoating was successfully prepared on the hydrofluoric acid etched zirconia surface using ALD. The subsequent annealing process transformed the TiO_2_ from amorphous to anatase and simultaneously generated a nanoscale topography. The resulting nanoscale structure was combined with the HF etched microscale structure to constitute an overall hierarchical micro/nanostructure. ALD-TiO_2_-A1 was annealed at 800°C for 5 min, and the treatment of short-time and high-temperature formed a regular and uniform wavelike nanostructure without altering its micromorphology. In contrast, ALD-TiO_2_-A2 was annealed at 300°C for 1 hour. The lower temperature but longer time induced the anatase TiO_2_ coating aggregate into irregular sphere-like structures, also shown in another study [52].

According to previous research reports, the cell-binding sites of proteins were more exposed on the surface of anatase, so the adsorbed proteins were better able to bind to the integrins, promoted cell adhesion and spread, and controlled the downstream osteoblast differentiation and mineralization [53]. Our study shows consistent results that hBMSCs grown on anatase TiO_2_ coated surfaces exhibit better cell adhesion, proliferation, osteogenic differentiation, and mineralization than those on HF and amorphous TiO_2_ coated surfaces. In addition, since the two anatase TiO_2_ nanocoatings have the same crystallinity, we suggested that the promoted osteogenic behavior on ALD-TiO_2_-A1 surfaces might be induced by its unique nanoscale topography via the canonical Wnt/*β*-catenin signaling pathway. However, the specific mechanism needs to be further studied and verified.

To sum up, the combination of an anatase TiO_2_ coating and an appropriate hierarchical micro/nanotopography synergistically promotes bone formation around zirconia implants in vitro and in vivo. The optimal annealing temperature and time and the possibility of a more suitable atmosphere need to be further investigated to achieve the best bone formation effect.

## 4. Conclusion

In this study, we developed TiO_2_ nanocoatings on microstructured zirconia surfaces using ALD. The subsequent annealing step transformed the crystal type of the deposited TiO_2_ nanocoating from amorphous to anatase and generated nanoscale structure simultaneously.

The TiO_2_ nanocoatings and annealing process did not change the microroughness of the zirconia surface, but the nanoscale topography differs depending on the annealing conditions. A regular wavelike nanopattern can be formed after annealing at 800°C for 5 min, and a nanoscale sphere-like structure can be generated after annealing at 300°C for 1 h.

The in vitro and in vivo results indicated that the anatase TiO_2_ coating with a regular wavelike nanostructure can promote bone formation around zirconia implants via the canonical Wnt/*β*-catenin signaling pathway, especially in the early healing stage. These findings suggest the proposed surface modification approach is promising for zirconia implants.

## Figures and Tables

**Table 1:** The primer pairs used in this study for qPCR analysis.

Gene	Forward (5ʹ-3ʹ)	Reverse (5ʹ-3ʹ)
*β*-actin	CCCAGAGCAAGAGAGG	GTCCAGACGCAGATG
ALP	CAACCCTGGGGAGGAGAC	GCATTGGTGTTGTACGTCTTG
Runx2	GTGCCTAGGCGCATTTCA	GCTCTTCTTACTGAGAGTGGAAGG
Col 1*α*1	GGGATTCCCTGGACCTAAG	GGAACACCTCGCTCTCCA
OSX	CCCCACCTCTTGCAACCA	GGCTCCACCACTCCCTTGTAG
Fzd4	CTCTGGCTCCCCTCATC	TCAAATACTGCACCGACCT
Axin2	TGAGCAACTGCGACAAAA	GCAGCATCTTCAATAGCCA
Lrp5	CGGCAGGACGTGTAAGG	AGCACGATGTCGGTGAAG
Lrp6	CAGGCCACCAATACAGTTG	CTCCCTTCATACGTGGACA

**Table 2:** Surface roughness.

Sample	Sa (*μ*m)	Sq (*μ*m)	Sz (*μ*m)
HF	1.30 ± 0.09	1.65 ± 0.13	11.37 ± 1.50
ALD-TiO_2_	1.43 ± 0.06	1.76 ± 0.08	11.39 ± 1.06
ALD-TiO_2_-A1	1.27 ± 0.12	1.62 ± 0.17	13.70 ± 1.73
ALD-TiO_2_-A2	1.38 ± 0.12	1.69 ± 0.07	10.52 ± 1.05

## Data Availability

The data used to support the findings of this study are available from the corresponding author upon request.
